# Correction to: Plasma Fibrinogen Is a Natural Deterrent to Amyloid β-Induced Platelet Activation and Neuronal Toxicity

**DOI:** 10.1186/s10020-019-0100-7

**Published:** 2019-07-30

**Authors:** Vijay K. Sonkar, Paresh P. Kulkarni, Susheel N. Chaurasia, Ayusman Dash, Abhishek Jauhari, Devendra Parmar, Sanjay Yadav, Debabrata Dash

**Affiliations:** 1 Department of Biochemistry, Institute of Medical Sciences, Banaras Hindu University, Varanasi, Uttar Pradesh India; 2 Indian Institute of Science Education and Research, Kolkata, India; 3 Developmental Toxicology Division, Indian Institute of Toxicological Research, Lucknow, Uttar Pradesh India; 4 Present address: Department of Biochemistry, School of Life Sciences, Central University of Rajasthan, Bandrasindri, Kishangarh, Ajmer, India; 5 Department of Internal Medicine, University of Iowa, Iowa City, USA

**Correction to: Mol Med 22:224–232, 2016**

**DOI 10.2119/molmed.2016.00003**

Following publication of the original article (Sonkar et al. 2016), the author reported an error in Fig. 1. The correct version of Fig. 1 is as follows:

**Fig. 1:**
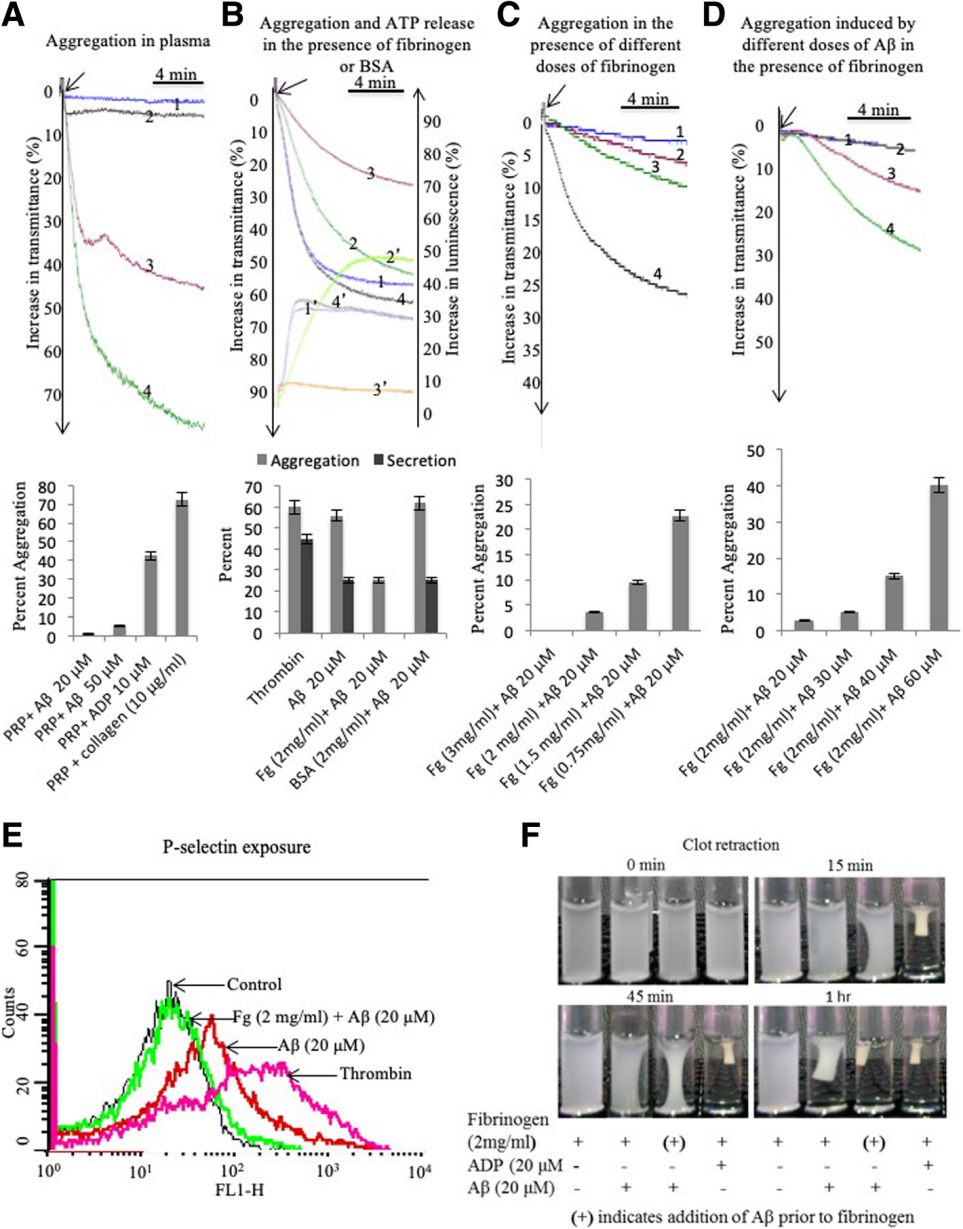
Effect of fibrinogen on Aβ_25–35_-induced platelet activation. **a** Tracings 1, 2, 3 and 4 represent platelet aggregation in PRP induced by Aβ_25–35_ (20 and 50 μmol/L), ADP (20 μmol/L) and collagen (10 μg/mL), respectively. The corresponding bar diagram is shown in the lower panel. **b** Tracings 1–4 represent aggregation, and tracings 1′–4′ represent corresponding dense granule secretion in washed platelets stimulated with either thrombin (1 U/mL; tracings 2 and 2′) or Aβ_25–35_ (20 μmol/L; tracings 1, 1′, 3, 3′, 4 and 4′). Tracings 3 and 3′ represent platelets pretreated with fibrinogen (2 mg/mL), whereas tracings 4 and 4′ represent platelets pretreated with BSA (2 mg/mL). Corresponding bar diagram is shown in the lower panel. **c** Tracings 1, 2, 3 and 4 represent aggregation of washed platelets induced by Aβ_25–35_ (20 μmol/L) in the presence of 3, 2, 1.5 and 0.75 mg/mL fibrinogen, respectively. Corresponding bar diagram is shown in the lower panel. **d** Tracings 1, 2, 3 and 4 represent aggregation of washed platelets induced by Aβ_25–35_ (20, 30, 40 and 60 μmol/L, respectively) in the presence of fibrinogen (2 mg/mL). Corresponding bar diagram is shown in the lower panel. **e** Flow cytometric analysis of expression of P-selectin on surface of platelets treated with different reagents and agonists as indicated. **f** Retraction of fibrin clot by platelets stimulated with either Aβ_25–35_ (20 μmol/L) or ADP (20 μmol/L) in presence of fibrinogen (2 mg/mL) added either before or after exposure to agonists as indicated
